# Perceived discrimination among Maghrebi users of health services in Tarragona (Spain)

**DOI:** 10.1186/s12939-015-0275-7

**Published:** 2015-12-03

**Authors:** Lourdes Rubio-Rico, Alba Roca-Biosca, Inmaculada de Molina-Fernández

**Affiliations:** Department of Nursing, Rovira i Virgili University, Tarragona, Spain; UNESCO Chair of Intercultural Dialogue in the Mediterranean, Rovira i Virgili University, Tarragona, Spain; Universitat Rovira i Virgili, Avda Catalunya, 35, 43002 Tarragona, Spain

## Abstract

**Background:**

Discrimination in health services for reasons of nationality or ethnicity is not a rare occurrence. This work aims to qualitatively analyse the perceived discrimination among Maghrebi community in Tarragona (Spain) with regard to the healthcare services they receive.

**Methods:**

A qualitative study was carried by means of 12 semi-structured interviews and 10 focus groups with Maghrebi adults living in Tarragona. The scope of the study was public health services in the area. A content analysis was performed using open coding.

**Results:**

Our results show that perceived discrimination is greater than actual discrimination because the deficiencies of the healthcare system are often interpreted as unfairness. However, our subjects also recounted incidents of clear discrimination against Maghrebi users of the healthcare system. The tendency to feel discriminated against is the culmination of an interaction between the group's low self-esteem and locals' often negative sentiments towards the group.

**Conclusions:**

We suggest addressing the shortcomings of the healthcare system in order to reduce this level of perceived discrimination and thus improve patient satisfaction. To improve this group's self-esteem and change how they are perceived, public policies should be put into effect which promote social inclusion and the respect for Maghrebis' rights as people, with actions taken on both fronts: in the host society and within the Maghrebi community itself. Furthermore, an active role for the patient with regard to his or her rights should be encouraged in order to minimize abuse from professionals and to facilitate institutional control of individual actions.

## Background

Discrimination has been recognized as a cause of health inequality [1, 2]. In fact, it has been shown that discrimination leads to a less than optimal use of healthcare services [3, 4] for a few different reasons. In addition to restricted access to public services, which is itself a form of discrimination, affective factors associated with the perception of discrimination result in self-imposed restrictions that affect access to and use of healthcare services [4–6]. It has also been shown that the perception of discrimination can have a negative effect on physical and mental health [7–14]; is associated with unhealthy habits and lifestyles [15]; affects the state of social determinants of health, such as working conditions and the degree of social support received [4, 7, 16]; and in the healthcare context, is closely linked to patient dissatisfaction [17].

Discrimination cannot be overlooked when assessing the relationships between healthcare institutions and healthcare system users from diverse geographical and cultural backgrounds. As health is a fundamental right [18], discrimination in a healthcare setting should be an exceptional circumstance. However, studies have shown that healthcare discrimination due to reasons of nationality or ethnicity is not that uncommon [6, 17, 19]. In fact, the 2006 Health Survey for Catalonia [20] (the most recent available version with queries about perceived discrimination) revealed that 7,2 % (95 % confidence interval [CI] 5,4-8,9) of foreigners in general, and 14.7 % (CI 10,5–18,8) of Maghrebi men and women experience feelings of discrimination when using healthcare services.

Maghrebis comprise 20.7 % of the foreigners registered in Catalonia [21] and, according to Navas and Cuadrado [22], locals tend to perceive them more poorly than any other immigrant group, disproportionally assess their relative numbers, and exaggerate perceived differences between their own culture and the culture of this group. Additionally, the Pew Report (2008) on global sentiments puts Spain at the top of the list of European countries in terms of people who most reject Islamic culture.

The current and very unfavourable opinion towards such a large group that often feels discriminated in the context of healthcare services, and the fact that this perceived discrimination can translate into negative health effects, justify this work, which aims to qualitatively analyse the experiences of discrimination felt by Maghrebi men and women in relation to public health services.

Finally, if perceptions of ethnic or cultural discrimination were to be identified in the public health services, this would oblige us to question the integrity of institutions that on one hand assert a commitment to fair and equal treatment of all users regardless of origin [23] whilst on the other hand may be permitting subtle acts of discrimination that cause certain users to feel discriminated against. Shedding light on this matter would help bring about the necessary changes between individuals and the institutions in question. Understanding the issues that foster discrimination would help to inform the decisions taken by politicians and professionals who are committed to improving quality and equality in the health service.

## Methods

### Design and setting

We used a qualitative design for descriptive and interpretive purposes.

The data were collected in semi-structured interviews and focus group sessions. The scope of the study was public health services in the area.

### Participants

The study population was considered to be adults (≥18 years) born in any of the countries of the Maghreb, residing in the province of Tarragona and with recent experience (≤6 months) of the health services in the area. To complete the spectrum of people with specific difficulties in relation to health services, we decided to include the testimony of two people who were not registered as residents and therefore had no regular access to the public health care system, despite having expressed the need to use it.

Purposive sampling was conducted until data saturation was achieved. The focus groups of adult patients were based on the information providers’ gender, cultural affiliation (Arab or Berber), level of education, language proficiency and rural o urban environment of origin.

Access to the Maghrebi adults took place in the community. The recruitment of information providers took place using the snowball technique, with people of Maghrebi origin initially accessible to the researcher, or through health and social work professionals who were in contact with the community, who were able to recruit individuals or groups of people from the study population.

### Data collection

The data were collected between May 2009 and May 2011 by means of 12 semi-structured interviews and 10 focus groups.

A pilot test was conducted in the first three focus group sessions, after which it was decided to include an external translator in all sessions with patients and to complete the oral consent with an informed consent form in Arabic.

As the data collection progressed, and in view of the need to complement the patient’s limited experience with another that was more extensive and comprehensive, the testimony of Maghrebi adults who were working as intercultural health mediators for the studied group was included in the research. The treatment of women as health agents par excellence among the Maghrebi community [24] suggested that an over-representation of women would be appropriate. Data saturation was accepted when new categories stopped appearing in the coding.

When assigning data collection techniques to the type of respondents, we decided that the mediators should be interviewed individually to gain an in-depth overview of the subject for study. The focus group technique was decided upon for patients, and to foster discussion. Some patients expressed their reluctance to participate in focus groups and others had difficulties in terms of time availability to attend the meeting. In both cases, they were offered the opportunity to participate in the study by means of an individual interview.

The individual interviews and focus group sessions were conducted by the lead researcher. In both cases, participants were asked to describe their experience with regard to the treatment they had received (or observed, in the case of mediators) from healthcare professionals and to reflect on the reasons behind that treatment. The topic of discrimination arose in almost all cases. This work is based on extracts from the data collected in relation to this topic.

The individual interviews were conducted at a venue proposed by the respondent. The focus group sessions took place in neutral spaces agreed upon by both parties or in family spaces for the participants, since many of the focus groups were groups that had previously been organized for other reasons (literacy, neighbourhood, university, etc.). The sessions were recorded in audio, and a literal transcript of the translated content was made. Twelve interviews were held, six with health service users and six with mediators, and ten focus group sessions were held with between three and eleven participants. The full details are shown in Table 1.

**Table 1:** Description of focus groups

ID. GROUP	Segmentation	Number of participants	Duration	Sex	Age: Mean (min - max)	Linguistic command: n (%)	Cultural affiliation: n (%)	Environment of origin: n (%)	Educational level: n (%)
FG1	Women. Arab. Poor linguistic command.	6	44'	Women: 6 (100 %)	34 (21–55)	None or low: 6 (100 %)	Arab: 6 (100 %)	Urban: 5 (83 %) Rural: 1 (17 %)	Illiterate: 2 (33.3 %) Literate: 1 (16.7 %) Primary ed.: 1 (16.7 %) Secondary ed.: 2 (33.3 %)
FG2	Women. Arab. University level.	3	137'	Women: 3 (100 %)	30 (25–37)	Basic: 2 (66.7 %) High: 1 (33.3 %)	Arab: 3 (100 %)	Urban: 2 (67 %) Rural: 1 (32 %)	Higher: 3 (100 %)
FG3	Women. Berber. Primary education not completed. Poor linguistic command. Rural environment.	7	77'	Women: 7 (100 %)	36 (27–42)	None or low: 7 (100 %)	Berber: 7 (100 %)	Rural: 7 (100 %)	Illiterate: 3 (42.9 %) Literate: 4 (57.1 %)
FG4	Women. Arab. Poor linguistic command.	5	46'	Women: 5 (100 %)	35 (27–41)	None or low: 5 (100 %)	Arab: 5 (100 %)	Urban: 1 (20 %) Rural: 4 (80 %)	Illiterate: 2 (40 %) Literate: 2 (40 %) Secondary ed.: 1 (20 %)
FG5	Women. Berber. Poor linguistic command.	4	58'	Women: 4 (100 %)	39.50 (28–55)	None or low: 4 (100 %)	Berber: 4 (100 %)	Urban: 3 (75 %) Rural: 1 (25 %)	Illiterate: 3 (75 %) Secondary ed.: 1 (25 %)
FG 6	Women. Arab. Primary and secondary education completed	5	55	Women: 5 (100 %)	28 (19–43)	None or low: 1 (20 %) Basic: 4 (80 %)	Arab: 5 (100 %)	Urban: 4 (80 %) Rural: 1 (20 %)	Primary ed.: 2 (40 %) Secondary ed.: 3 (60 %)
FG7	Women. Arab. Primary and secondary education completed. Urban environment.	3	52	Women: 3 (100 %)	28 (27–32)	None or low: 2 (66.7 %) Basic: 1 (33.3 %)	Arab: 3 (100 %)	Urban: 3 (100 %)	Primary ed.: 2 (66.7 %) Secondary ed.: 1 (33.3 %)
FG8	Mixed. Poor linguistic command. Urban environment.	9	91	Women: 6 (66.7 %) Men: 3 (33.3 %)	26 (22–37)	Basic: 9 (100 %)	Arab: 5 (55.6 %) Berber: 4 (44.4 %)	Urban: 9 (100 %)	Primary ed.: 3 (33.3 %) Secondary ed.: 3 (33.3 %) Higher: 3 (33.3 %)
FG9	Mixed. Poor linguistic command. Urban environment.	4	96	Women: 1 (25 %) Men: 3 (75 %)	28.5 (24–45)	Basic: 4 (100 %)	Arab: 3 (75 %) Berber: 1 (25 %)	Urban: 4 (100 %)	Primary ed.: 1 (25 %) Secondary ed.: 2 (50 %) Higher: 1 (25 %)
FG10	Women. Poor linguistic command.	11	67	Women: 11 (100 %)	43 (24–52)	None or low: 11 (100 %)	Arab: 6 (54.5 %) Berber: 5 (45.5 %)	Urban: 10 (91 %) Rural: 1 (9 %)	Illiterate: 8 (72.7 %) Primary ed.: 1 (9.1 %) Secondary ed.: 2 (18.2)

### Data analysis

An analysis of the content was performed. The first level of analysis was concurrent with the data collection and aimed to identify emerging themes to modify the sampling and data collection work. In the second level, the data were segmented and the reporting units were identified, and coded using open coding. The categories that emerged were integrated into a higher level of organization based on the properties and dimensions of a single concept.

The interpretation of the data sought to establish relationships between different levels of organization of the content, either between categories, between concepts or between categories and concepts.

To ensure the validity of the analyses, the study relied on the collaboration of two researchers specializing in health and immigration, as well as two health professionals with experience in the care of Maghrebi patients. Initially, a joint analysis was carried out between the lead researcher and the health and immigration specialists. Then the preliminary analysis was submitted so that healthcare professionals with experience working with the group under study could assess its veracity and coherence.

The systematization of the categorization and analysis was performed using the program ATLAS-ti WIN version 5.0.

### Ethical considerations

Although this project did not focus on any healthcare institution and did not recruit any patients, but instead adults with experience as patients of health services or with a perceived need to use that service, all the collaborating institutions had prior access to the project and gave their approval. Furthermore, to comply with the ethical requirements of research, an informed consent document was produced. This document was translated into Arabic in a sworn translation and from the point when it became available (February 2010), it was accepted and signed by all participants able to read and write in that language. The participants in the study who were not literate in Arabic or who had provided testimony before the consent document was available gave their oral consent after being informed of the same aspects as those listed in the written consent.

## Results

The sociodemographic characteristics of the participants are shown in Table 2.

**Table 2:** Sociodemographic characteristics of respondents

		Users		Mediators		Total
Age in years: mean (CI 95 %)	35 (33–38)		39 (30–47)		36 (33–38)	
Duration of the interview in minutes: mean (CI 95 %)	69' (63'–75')		76' (57'–95')		70' (64'–75')	
	n	%	n	%	n	%
Sex						
Man	9	14.3 %	1	16.7 %	10	14.5 %
Woman	54	85.7 %	5	83.3 %	59	85.5 %
Year of arrival						
Before	2	3.2 %	0	0.0 %	2	2.9 %
1995–1997	6	9.5 %	1	16.7 %	7	10.1 %
1998–2000	5	7.9 %	0	0.0 %	5	7.3 %
2001–2003	13	20.6 %	2	33.3 %	15	21.7 %
2004–2006	18	28.6 %	3	50.0 %	21	30.4 %
2007–2009	14	22.2 %	0	0.0 %	14	20.3 %
2010	5	7.9 %	0	0.0 %	5	7.3 %
Type of interview						
Focus group	57	90.5 %	0	0.0 %	57	82.6 %
Individual	6	9.5 %	6	100.0 %	12	17.4 %
Cultural affiliation						
Arab	39	61.9 %	5	83.3 %	44	63.8 %
Berber	24	38.1 %	1	16.7 %	25	36.2 %
Environment of origin						
Rural	21	33 %	2	33 %	23	33 %
Urban	42	67 %	4	67 %	46	67 %
Linguistic command						
None or low	41	65.1 %	0	0.0 %	41	59.4 %
Basic	19	30.2 %	0	0.0 %	19	27.5 %
Advanced	3	4.8 %	6	100.0 %	9	13.0 %
Level of education						
Unable to read or write	20	31.7 %	0	0.0 %	20	29.0 %
Able to read and write	7	11.1 %	0	0.0 %	7	10.1 %
Primary educ.	13	20.6 %	0	0.0 %	13	18.8 %
Secondary ed.	15	23.8 %	1	16.7 %	16	23.2 %
University	8	12.7 %	5	83.3 %	13	18.8 %
Experience of care						
Primary and hospital	55	87.3 %	5	83.3 %	60	87.0 %
Primary	6	9.5 %	1	16.7 %	7	10.1 %
No regular access	2	3.2 %	0	0.0 %	2	2.9 %

The results were divided into three content groups: the different ways in which perceived discrimination occurs, *The faces of discrimination;* users’ positions when judging incidents that could be interpreted as discriminatory: *About feeling discriminated against…or not*; and the reasons attributed to that feeling: *The whys.*

The transcriptions were annotated according to the following model: type of data collection activity (II/FG), order number of the data collection unit, and a distinction between users (U) and cultural mediators (CM).

### The faces of discrimination

In the reports describing perceived discrimination, three categories emerge: unmet expectations, very clearly reprehensible professional activities which are not strictly attributable to the differential treatment of Maghrebi users, and the objectively discriminatory undervaluation of Maghrebi users.

#### Unmet expectations

Waiting to receive attention, the poor conditions of the hospital rooms they were admitted to, difficulties resolving certain health problems, the perception of cold treatment, very short visits, even the use of certain biological protective measures, such as gloves, is perceived as specific to immigrants and identified as discrimination:

Well…with a foreigner who has a cold they use gloves and every precaution, but with somebody from here they act normal, as if nothing were the matter (FC9).

The room occupancy policy in effect in some hospitals, often based on a foreigner-national division, also generates perceived discrimination:

Foreigners with foreigners; Spaniards with Spaniards.

-Yes, yes.

-But H. was with a gypsy.

-Yes, yes, here gypsies are also treated like foreigners, and they won’t put one [a gypsy] with a Spaniard or Catalan (FC2).

Such situations serve as a pretext for thinking about discrimination beyond the healthcare sector. The underlying idea is the contradiction between the message that the rights of citizens are acquired through the fulfilment of civil duty and the fact that a person’s origin apparently determines their rights as a citizen:

So as not to feel like a second class citizen, politicians claim that we are all citizens. If I want to operate a business I have to do everything that Catalans or Spaniards do here. I have to follow corporate laws…just like everyone else! Catalan! I also pay taxes the same way, so why do they get taken to one room and me to another? (FC2).

#### Very clearly reprehensible professional activities

This group includes professional activities which are definitely reprehensible but that do not always pertain directly to the notion of differential treatment towards Maghrebis, although they may easily be experienced as discriminatory:

Q: Have you felt discriminated against for being foreigners?

Yes, yes, yes, it has happened, yes. […] I had kidney problems and I was hospitalized. I asked the nurse to help me go to the bathroom and she said, […] ‘and where is your mother?’ I said, ‘Well… my mother went home,’ and she said, ‘Well this is something you’ll have to ask her to do.’

Q: Do you believe it was because you’re a foreigner?

Yes, I think so. Because that was the first…but when the doctor came in she changed her tune (II3U).

#### Demonstrable discrimination

While patients do not always feel like they have been victims of objectively unequal treatment, based on their stories, we have been able to identify reprehensible professional activities motivated by the user’s place of origin:

When there’s a foreigner, they’re treated like crap, and they’re looked at like… there are some who… doctors as well as administrative staff… at times, depending on who the person is… there’s a different behaviour… they say to your face, ‘Go back to your country!’ That’s it, that’s what I’ve seen! (FG9).

Professionals might also express disagreement with a certain way of doing things, without medical justification for what is said or how it is presented:

And when you go to this [doctor], he always says things like, ‘Why do you wear the veil? Why do you dress like this? Why....?’ Whenever a person comes in with this [touching her headscarf] he always bring up the same topic. During an ultrasound visit, when he saw it was a baby girl, he said ‘It’s a girl, but without a scarf.’ Do you understand? ‘A girl, but without a scarf’ And later, ‘A girl will be born here, and when she grows up you’re not going to make her wear a hijab.’ This happens every time! Every time! (FG2).

The perception of discrimination among Maghrebi women is often associated with experiences in which professionals feel entitled to undervalue or criticize them simply for displaying characteristics which fit in with their stereotypes:

She says there is a gynaecologist who is very hard to deal with and always says, ‘You already have enough kids’ to all Maghrebis, and ‘You shouldn’t have so many kids’ You feel pressured, you feel bad (FG5).

When I don’t wear a headscarf and go to the hospital everything is fine, everything is said nicely…but when I wear a headscarf there is a nurse with somebody at the counter, and she doesn’t look at me while speaking and sometimes even shouts. Sometimes she comes and goes and acts like I’m not even there. But it’s not everybody. There are some very good people and others who are very racist (FG6).

The perception of discrimination progresses to the point that certain professional actions could be seen as a restriction of rights specifically aimed at Maghrebi patients:

My finger hurts, I don’t know how to speak [Spanish] but [I do speak] French, and they told me, ‘Go and learn Spanish and I’ll learn Arabic.’ (FG6).

Moreover, certain discriminatory practices are known and repeated by other professionals. There is one case of a doctor who, according to the mediator, repeatedly hid the gender of the foetus from pregnant Maghrebi women:

When I went to the hospital for an interview, a lady told me, ‘Do you know what a doctor did to me?’ and I tell them, ‘I already know. You don’t need to tell me his name.’

Q: What’s this person’s attitude?

Total rejection of immigrants, especially Moroccans. The other day in a workshop on gestational diabetes, there were three Moroccan women who were at different stages of pregnancy and all of them knew if they were carrying a boy or a girl. Only one woman didn’t know…she told me ‘The doctor didn’t want to tell me the sex of the baby.’ Looking at her, without saying anything, I already knew what neighbourhood she was from. I then asked her in Arabic, ‘Do you live in [says the name of the area where this professional works]?’ She replied: ‘Yes!’ (II10CM).

In short, we can say that Maghrebi patients feel that they experience discrimination more frequently than it effectively occurs, despite having identified objectively discriminatory practices aimed at Maghrebi patients based on national, social, or cultural arguments with little to no relation to health.

### About feeling discriminated against…or not

In general, patients who state that they have felt discriminated against also identified, from a range of experiences with various professionals, experiences which were not discriminatory, or which were even excellent, thus attesting to the notion that the perceptions of the patients towards the attitudes of healthcare professionals is not premeditated. ‘They’re not racist here. They look at the person. It’s not everyone; most people are not racist towards the headscarf’ (FG1).

Though dissatisfactory experiences are a major source of perceived discrimination, some patients do not see them as discriminatory treatment and instead identify them as deficiencies in the system that affect all patients alike:

Yes, it’s the same for everyone. You can be speaking to someone but when you go outside somebody else (a local) tells you ‘Look, my appointment’s a very long way off.’ And you think ‘Wow, she has to wait longer than me.’ It’s normal. Regardless of whether you’re Spanish or a foreigner, it’s the same (FG7).

With similar reasoning, another group of patients states that they have not felt discriminated against and bases this assertion on the humanity of the treatment and the quality of the service received. They report receiving service as good as that received by local patients:

I had my daughter here and it was just like everyone else, and afterwards I had three miscarriages and they treated me the same. I never feel like I’m from somewhere else, you know what I mean? Never! And they treat me very well, and equally. Also, when I take my daughter to her doctor the nurses know me and greet me… (FG4).

Or as bad…because it appears there are some professionals who treat everyone just as poorly, regardless of their origins…locals and immigrants alike:

My GP treats me the same as everyone else. If he doesn’t smile, then he doesn’t, it’s how he is. When calling out the names he always has the same face, like this [imitates the unpleasant face] (FG2).

This is confirmed by another participant in the study, who recounts an incident experienced as the assistant to a Spanish woman, who she looks after professionally:

I went with the woman I take care of. Spanish. Yes. She got very mad with him [the doctor]. He didn’t listen to her, didn’t listen. One day she almost fought with one. It was the eye doctor. She wanted to say the medications she was taking, and he wouldn’t let her speak (FG4).

Finally, some cautiously interpret the experiences of dissatisfaction, alleging that despite feeling disappointed by the situation experienced, they would not go so far as to attribute it to premeditated discrimination against Maghrebis because they are unfamiliar with the experience of other users of the healthcare system:

At times, it takes a very long time until my appointments with my GP and I don’t understand […] I don’t know if it’s because I’m a foreigner because I have no way of knowing (FG6).

In any case, the participants generally agreed that their assessments of their experiences, both good and bad, reflect the personal position of the staff member:

I think it has more to do with the person than with the fact that I’m an immigrant. What I’ve noticed is a few things in my experiences that I think are because I’m an immigrant, but not many. […] It depends on the person (FC2).

In general, users believed that despite the fact that institutions issue certain guidelines regarding the professional-patient relationship, it is possible to become a healthcare professional without strong human relations skills:

In theory, all professionals already study this part of…psychology, but it seems like they need to be more restrictive in their selection of people. They need to take it more seriously. (FG2).

And there is no institutional control over the actions of professionals, so that once they get a job they are free to do whatever they want. In light of this arbitrariness, the patient may feel discriminated against:

A friend went to get a health card and had some problems the first time. He went back and everything got resolved. So…I think that if he has the right for these matters to be resolved, [the professional] will resolve them when he goes there the first time without any problems or commentary. What is this? Doesn’t he like to resolve such things? I get the impression that there’s… What’s it called? Racism. They don’t like foreigners (FG9).

### The whys

Some contributors noted a certain tendency for Maghrebi patients to see discrimination and racism when the problem was actually difficulties inherent to being and feeling like an immigrant, or problems in figuring out how the system works and in understanding other people.

One of the interviewed mediators explains it as follows:

Being an immigrant is very difficult… and if you aren’t an immigrant you can’t understand what it’s like. When you’re an immigrant you notice it, and as one myself I understand what these Moroccans are experiencing: it’s an interpersonal conflict people have: ‘People here don’t understand me.’ They’re not in their country… (II11CM).

Users of the healthcare system also recognize a certain tendency to identify negative experiences with professionals or the system itself as discriminatory treatment:

We cannot say that it [a bad experience with a professional] always happens, but as I’m an immigrant, when she [the professional] says this, the first thought is that it’s because I’m an immigrant and that it wouldn’t happen if I were Spanish (FG2).

The most common causal attributions associated with this feeling are the person’s place of origin and the fact that he or she is not a national:

Some [Moroccans] feel, like, inferior when a professional asks for things. They say, ‘Yes, they’re racist because I’m Moroccan’ (II11CM).

Difficulties expressing themselves:

If they see that it’s difficult for you to express yourself, they make you feel like a nuisance and they don’t make an effort. They must think it’s just your problem. If I try to explain myself and I have troubles I’m told ‘I can’t waste my time, I have a lot of people waiting’ (II6).

There is also an awareness of cultural and social differences, especially with regard to physical appearance and clothing:

I think that there are professionals who think, ‘These people are very closed, they don’t let me look at them or lift their jumper to look at their stomach.’ You know what I mean? You get this feeling about yourself… you feel under… undervalued. It could be that they treat us Moroccans differently because they don’t know us well, and they don’t treat us like the people who are from here and who dress like them. It makes you feel bad about yourself (II7U).

But, how did this perception emerge? We have identified two determining factors. On the one hand, there seems to be an internalized feeling of unworthiness for the benefits to which citizens are entitled:

It’s as if Maghrebi women think that they can’t make any requests or complaints, you know? (II7U).

On the other hand, there is the image which the host society in general and health service workers in particular project onto Maghrebis. This image portrays them as professional beggars for the benefits they are entitled to by law:

When they’re talking about the problem of immigration they speak as if it’s a negative point in two regards: taxes − they say they don’t pay taxes, and healthcare − they say they’re always at the health centre [conversation continues with complaints from others]. These are the things they say, even in the media, they talk about immigrants. I’m an immigrant! (FG2).

[…] because [for] my daughter, I need the strips, one day I need needles, another I need insulin, and do you know how I feel? Weak. As though they see me as always asking for something, you know? (FC4).

The internalization of an eroded self-esteem and input from the outside creates a vicious cycle. Accordingly, the damaged self-esteem seems to reinforce the characterization of being ‘the other’ and as having less value and, in turn, this negative characterization encourages the representation of oneself as an unworthy person. All of this feeds the perception of being discriminated against.

## Discussion

This analysis has revealed that the perception of discrimination among Maghrebi health service users is more common than actual discrimination. Perceived discrimination is closely associated with experiences of dissatisfaction, and is not always the result of differential treatment. Nevertheless, situations have been identified in which Maghrebi patients are discriminated against for cultural, linguistic, or ethnic reasons. Our informants perceive the attitudes of healthcare professionals towards Maghrebis in general as arbitrary.

With regard to the limitations of this study, it is worth mentioning that it was not possible to organize an entirely male focus group. Men took part as individual informants or as part of mixed focus groups. The possibility cannot be ruled out that an entirely male focus group would have contributed new insights to this discussion.

Furthermore, the data collection techniques only allowed us to identify perceived discrimination. Without directly observing acts of discrimination we were unable confirm whether it was actually present. Finally, given that the respondents’ declarations referred to events that had occurred at least six months previously, we cannot rule out the possibility that their recollections of these events may have become distorted over time.

Among the incidents the respondents perceived as discriminatory, a distinction was not always drawn between systemic shortcomings and the reprehensible actions of professionals as opposed to actual differential treatment towards Maghrebi patients. However, it is worth pointing out that discrimination does not have to be objective for it to affect individuals’ health. On the contrary, simply the perception of discrimination can have negative health repercussions [6, 7, 11, 25]. However, the cases in which there was clear mistreatment specifically aimed at Maghrebi patients should not be understood as a subjective problem, as the discrimination there was evident.

Some studies have identified the communicative style used by health professionals as a clear source of perceived discrimination [26, 27]. The participants in the present study confirmed this notion and spoke of verbal and non-verbal expressions that communicated rejection.

Regarding the linguistic differences between health professionals and Maghrebi patients, it is worth emphasising that these reinforce the two ways that perception of discrimination is generated. On the one hand, patients can feel at fault for not being able express themselves competently in one of the two official languages used in Catalonia and this can lead to low self-esteem. On the other hand, the health professional may feel that they have a right to look down on patients who cannot speak their language, which in turn leads them to project the sense that Maghrebi men and women do not deserve the same level of care that is given to other patients. Linguistic differences obviously complicate the process of providing medical attention and mean that extra time and translation services must be factored in when planning for such patients. It goes without saying that the managers of healthcare institutions need to take such considerations into account. However, given that it is impossible for the system to provide language support at all times and under all circumstances, linguistic differences provide an excellent opportunity for health professionals to use their communication and interpersonal skills to show empathy and to provide care for patients who have difficulties communicating effectively [28, 29].

Discussions with the respondents show that the experience of discrimination in the context of healthcare services is not unusual. Quantitative studies in this area have found that the perception of discrimination in health services is more prevalent among this group than among foreigners in general [20]. This information may be alerting us to two facts, which are not necessarily mutually exclusive and which are very important in terms of caring for the health and well-being of the people affected. As a subjective perception, it may be an expression of the damaged self-esteem of a group which (and this is the second issue) is not highly valued by the local population [22, 30, 31]. The results of this work are congruous with these notions in that they recognize the lack of self-esteem and the rejection experienced by Maghrebi men and women as elements involved in the genesis of such perceived discrimination. All of this calls for action addressing both issues, as one reinforces and builds upon the other. Achotegui [12] suggests psychological and emotional education to allow people to successfully handle these kinds of difficulties. However, because the behaviour of the host society affects Maghrebi men and women, it seems clear that public policies for social inclusion and respect for the rights of people, regardless of their origin, can be considered basic strategies for maintaining the health of different immigrant groups [16, 32–34] and, quite possibly, improving their ability to live in peaceful coexistence within the host society.

Analysis of the testimonies we gathered reveal that similar situations are perceived differently. The causal attribution of an event is determined more by subjective appreciation than by an objective assessment, which would make it possible to differentiate between the shortcomings of the system, which cause feelings of dissatisfaction, and premeditated actions against the group, which cause feelings of being discriminated against. Whatever the case, and without rejecting the possibility that in certain cases negative attitudes are directed specifically at Maghrebi users, many of the situations identified by these patients as discriminatory also affect other users of the healthcare system. Satisfaction surveys among the general population indicate that the main reasons for dissatisfaction are waiting times [35–38], organizational aspects and bureaucratic issues related to healthcare [35, 39], the quality of the facilities [38], treatment from staff, and the clinical information received [36], without, of course, these patients interpreting such things as being the result of their place of origin, so perceived discrimination associated with dissatisfaction requires action to improve the shortcomings of the system.

As some of the respondents indicated, it is ultimately the healthcare professionals themselves who decide the extent to which they will involve themselves in the well-being of their patients. How can it be explained, then, that healthcare professionals choose to act differently in the same circumstances, with some deciding to ignore the fate of certain patients? It cannot be left to personal choice; it is a matter of professional obligation. At this point we must bring up the concept of moral disengagement. The term, which arose from the social cognitive theory of Albert Bandura [40], refers to the change in moral identity experienced by some people from a commitment to certain values to actions that go against those values. This is a gradual process which may go unnoticed by people and institutions until it is firmly established and comes to be perceived as normal [41]. This would explain the institutional passivity which some patients complain about in light of the clearly reprehensible actions of some professionals. The experiences which attest to the absence of a moral bond in some professionals in their relations with Maghrebi patients allow the mistreatment of this type of user, which professionals feel authorized to do, to be interpreted as the perception of an added demand for medical attention. It is as if the patient has to ask for forgiveness for the trouble he or she is causing, the expense generated or, even worse, for the fact that he or she is an immigrant, as suggested by Swahnberg, Hearn and Wijma [42], who identified foreigner status as a risk factor for abuse in healthcare settings.

According to Bandura [43], the dehumanization of the affected party is a requisite for moral disengagement. It is an unavoidable step in making suffering invisible and, if there is no suffering, the whole series of tolls patients have to pay in the form of various types of abuse does not raise any alarm. Witnesses who speak of clearly reprehensible actions aimed specifically at Maghrebi patients force us to ask ourselves if immigrant status, combined with being a woman in a group subject to widespread social rejection, further encourages morally disengaged professionals to dehumanize these people. Obviously, it would be interesting to study how these professionals address patients who believe that they have every right to receive proper medical treatment, who have mechanisms for defending themselves against abuse, and who actively work to defend their dignity as people, because the results of this study suggest that the more stripped of power a patient is, the more detached and less morally healthcare professionals behave towards that patient.

The variety of experiences that a single patient has in his or her relations with healthcare professionals and institutions indicates a discretionary attitude on the part of those professionals in their relationships with Maghrebi patients, a lack of administrative control over the individual actions of healthcare professionals, and an inconsistency between the institutions’ message of integration [23] and the specific actions of some professionals. All of this calls for intervention in terms of the professionals themselves as well as the healthcare system as a whole.

First of all, new research should look into the underlying factors which contribute to the nature of the relationships between healthcare professionals and patients. That is, it has to be determined whether the origin of the problems lies in the structure of the healthcare system itself or whether the objectionable actions documented in this work are due to dehumanized personal stances or attitudes of rejection towards immigrants or specific groups in particular.

The structural faults in the system leave patients feeling dissatisfied and even discriminated against. Likewise, the same difficulties may be conditioning the attitudes of health professionals towards patients that pose additional social, cultural and communicative challenges. In such circumstances, health institutions should implement strategies to ensure the equal provision of good quality healthcare.

With regard to individual attitudes, a recent study by Soler et al. [44] found medical students to be prejudiced in their handling of cultural diversity from the outset of their studies, despite having grown up in a multicultural society. To counter this tendency, the authors of the article underlined the need to introduce the concepts of health inequalities and intercultural skills to medical degrees [44], particularly given that respect for the independence, beliefs and culture of patients has been defined as a specific competence to be acquired during medical training in Spain since 2008 [45]. Although these aspects are already present in medical training, the results of our study demonstrate that they also need to be fostered amongst healthcare professionals who are already practicing.

The abusive professional practices reported, if confirmed, would represent an excessive challenge for this work, because as examples of dehumanized practice they would need to be analysed and the institutions implicated would need to be involved in addressing them. Nevertheless, it should be stressed that such actions are, if true, totally unacceptable, especially if they are known and tolerated, as indicated by some of the respondents. The health service cannot, under any circumstances, set out a series of regulations [23] without ensuring that they are complied with, as currently seems to be the case. Health institutions should play an active role in ensuring that all patients treated with the same level of respect. Actively questioning, without coercion, all the parties involved would allow us to determine the precise nature of relations between health professionals and patients.

On the other hand, active patient education in which they learn that they are obligated to certain duties and entitled to certain rights is essential to reverse the hierarchical relationship that is often established in the healthcare setting, and to prevent abuses of power in an already asymmetrical relationship. In this context, being an active user means being aware of the rights and duties that one has as a user of health services, fulfilling the obligations assumed as a result thereof, and knowing the mechanisms in place to make oneself heard, as well as being able to use those mechanisms when necessary. The promotion of active healthcare users and their rights will minimize abuse by professionals, in addition to promoting and facilitating institutional control over clearly reprehensible individual actions. Only if there is unequivocal evidence of misuse or abuse should the institution be obligated to take part in its resolution.

## Conclusions

Discriminatory perceptions among Maghrebi users are more common than actual discrimination and are based on the internalization of an eroded self-esteem and negative input from the outside. To improve this group’s self-esteem and change how they are perceived, public policies should be put into effect which promote social inclusion and respect for Maghrebis’ rights as people. To this end, it is essential that such actions are taken in both fronts; that is, in the host society and whitin the Maghrebi community itself.

To reduce levels of perceived discrimination and abuses by health professionals, the health service needs to provide the necessary resources that would enable clinical exchanges between health professionals and Maghrebi patients to be conducted in optimum conditions. Health professionals should also be encouraged to reflect on respect for the independence, beliefs and culture of patients.

The health service needs to actively monitor the individual actions of its professionals in order to protect the dignity of individuals when they come into contact with the service and to encourage patients to play an active role in exercising their rights.
